# Bioinformatics Analysis of Ferroptosis-Related Driver Genes in Stanford Type A Aortic Dissection

**DOI:** 10.3390/cimb48040382

**Published:** 2026-04-07

**Authors:** Ruizhi Nie, Weiqing Han, Jianjun Xu

**Affiliations:** The Second Clinical Medical School, Nanchang University, Nanchang 330006, China; 360014230010@email.ncu.edu.cn (R.N.); 360014240113@email.ncu.edu.cn (W.H.)

**Keywords:** ferroptosis, Stanford type A aortic dissection, bioinformatics analysis, ferroptosis-related driver genes

## Abstract

Stanford type A aortic dissection (TAAD) is a life-threatening cardiovascular condition associated with high mortality. Ferroptosis has been implicated in TAAD pathogenesis, but comprehensive analyses and experimental validation of ferroptosis-related driver genes (FRDGs) remain limited. This study systematically investigated FRDGs in TAAD using bioinformatics and experimental approaches. Differentially expressed ferroptosis-related driver genes (DEFRDGs) were identified by integrating the GSE153434 dataset with the FerrDb database. Functional enrichment analysis was subsequently performed, followed by the construction of a protein–protein interaction (PPI) network, assessment of immune cell infiltration, and prediction of potential miRNA interactions. Candidate hub genes were then validated using an independent cohort (GSE52093) and clinical tissue samples, with their diagnostic value evaluated via receiver operating characteristic (ROC) curve analysis and their protein expression confirmed by immunohistochemistry. We identified 25 DEFRDGs (17 upregulated, 8 downregulated) enriched in oxidative stress, iron binding, and ferroptosis/HIF-1 signaling pathways. Six hub genes (HIF1A, IL6, TIMP1, SAT1, HMOX1, LPCAT3) were significantly upregulated in validation cohorts, five genes (HIF1A, TIMP1, SAT1, HMOX1, LPCAT3) achieved an area under the curve (AUC) of 1.000, while IL6 also exhibited high diagnostic accuracy (AUC = 0.914). Fibroblast infiltration was elevated in TAAD tissues. Further miRNA interaction prediction revealed the potential involvement of miRNAs, such as miR-138-5p, miR-18b-5p, miR-199a-5p, miR-185-5p, miR-506-3p and miR-4644. Immunohistochemistry confirmed increased protein expression of HIF1A, SAT1, and LPCAT3. These three genes emerge as key ferroptosis-related drivers in TAAD. Their consistent upregulation and strong diagnostic performance support ferroptosis as a potential therapeutic target and provide a basis for mechanism-focused interventions.

## 1. Introduction

Aortic dissection (AD) is a catastrophic cardiovascular emergency characterized by its fulminant onset and high lethality [1]. Among its subtypes, Stanford type A aortic dissection (TAAD) represents the most severe and prognostically ominous form [2], associated with exceedingly high risks of aortic rupture and mortality [3,4]. The primary pathological event in TAAD is blood entering the aortic wall through an intimal tear, which creates true and false lumens [5]. Currently, emergency open surgical repair remains the most effective treatment for TAAD [6]. However, the procedure is highly complex, carries a significant risk of postoperative complications, and thus presents a formidable clinical challenge [7,8,9]. Although advancements in imaging and surgical techniques have contributed to a reduction in in-hospital mortality, the overall prognosis remains severe [10,11]. Furthermore, targeted pharmacological agents capable of effectively delaying, halting, or reversing the dissection process are still lacking [12]. Major pathological contributors to TAAD include endothelial dysfunction, phenotypic switching and apoptosis of vascular smooth muscle cells (VSMCs), fragmentation of elastic fibers, degradation of the extracellular matrix (ECM), and infiltration by activated immune cells [13,14,15]. Understanding the underlying mechanisms that govern these biological processes is crucial for the advancement of novel preventive and treatment interventions.

Ferroptosis is a novel form of regulated cell death that hinges on the accumulation of iron, resulting in heightened levels of iron, diminished glutathione reserves, and the oxidation of lipids [16,17]. It is often associated with distinct changes in mitochondrial shape such as reduced or lost cristae and a larger membrane density [18,19]. New evidence demonstrates ferroptosis as a driver of vascular injury and inflammation [20,21]. Extensive research has established its significant role in a wide array of cardiovascular diseases, including atherosclerosis [22], ischemia–reperfusion injury [23], doxorubicin-induced cardiotoxicity [24], stroke [25], heart failure [26] and aortic dissection [27]. A recent study demonstrated that the ferroptosis inhibitor BRD4770 alleviated aortic dilation in a mouse model of TAAD [27]. Immune cell infiltration is closely associated with the process of ferroptosis [28], suggesting a potential key role in TAAD pathogenesis. Inhibition of ferroptosis could be a promising therapeutic approach [22]. However, current research on ferroptosis in TAAD has largely focused on the regulation of individual genes and neglected systematic analysis of ferroptosis-related driver genes (FRDGs) and experimental validation.

Because of this gap in knowledge, we are interested in conducting a biological study of FRDGs in TAAD. We conducted a bioinformatic analysis using publicly available data sets to determine their roles and regulatory networks. Our specific objectives were to: dissect the association between FRDGs and TAAD pathogenesis; identify their underlying functional mechanisms and pivotal genes; and perform preliminary validation using clinical TAAD tissue samples. Our objective is to identify potential predictive genes for TAAD and provide a new perspective for the study of its pathogenesis.

## 2. Materials and Methods

### 2.1. Arterial Tissue Collection

The Biomedical Research Ethics Committee of The Second Affiliated Hospital of Nanchang University approved all protocols involving human specimens. Written informed consent was secured from every patient or their legal guardian prior to inclusion in the study. Aortic wall tissues for the analysis of TAAD were acquired from patients undergoing surgical repair for the condition. The study excluded individuals with connective tissue disorders such as Turner’s, Loeys-Dietz, Ehlers-Danlos, and Marfan’s syndrome [29]. Normal aortic wall tissues were collected from the unused portions of aortas discarded following cardiac transplantation surgery. Immediately after resection, all human specimens were promptly stored in liquid nitrogen or tissue fixative solution. All procedures involving human participants were carried out following the ethical standards of the institutional research committee [30].

### 2.2. Data Sources

Data were obtained from the Gene Expression Omnibus (GEO) database (https://www.ncbi.nlm.nih.gov/geo/ (accessed on 1 May 2025) [31]). We used the GSE153434 [32] dataset as the discovery cohort to identify differentially expressed genes (DEGs). This dataset comprises whole-transcriptome profiles from 10 ascending aortic samples of patients with TAAD and 10 normal ascending aortic samples. The GSE52093 [33] dataset, comprising data from 5 TAAD and 7 normal ascending aortic samples, served as an independent validation cohort for the candidate hub genes.

### 2.3. Identification of DEGs and DEFRDGs

DEGs were screened from the GSE153434 dataset using the GEO2R (https://www.ncbi.nlm.nih.gov/geo/geo2r/ (accessed on 1 May 2025) [34]) online analysis tool. The significance threshold was set at an adjusted *p*-value < 0.05 (Benjamini and Hochberg method) and an absolute log2 fold change (|log2FC|) > 1. This analysis yielded 1777 DEGs, including 688 upregulated and 1089 downregulated genes. Subsequently, differential gene expression analysis between the TAAD and healthy control groups was performed, with the results visualized using Principal Component Analysis (PCA) and volcano plots.

FRDGs were obtained from the FerrDb database (http://www.zhounan.org/ferrdb/current/ (accessed on 2 May 2025) [35]). After removing duplicates, a final set of 264 unique FRDGs was obtained. The overlap between the DEGs and FRDGs was then analyzed using R software (v4.2.1) to identify differentially expressed ferroptosis-related driver genes (DEFRDGs). We used the ggplot2 (v3.4.4) and VennDiagram (v1.7.3) packages to generate a Venn diagram showing 25 overlapping DEFRDGs. The expression pattern of these DEFRDGs across samples was visualized as a heatmap using the ComplexHeatmap package (v2.13.1).

### 2.4. GO and KEGG Enrichment Analysis

Enrichment analyses were conducted on the 25 DEFRDGs to explore their functional significance. The clusterProfiler package (v4.4.4) in the R programming environment was employed for this purpose. Gene Ontology (GO) and Kyoto Encyclopedia of Genes and Genomes (KEGG) pathways were analyzed to uncover enriched biological functions and pathways associated with the DEFRDGs. Enrichment terms with a statistically significant *p*-value < 0.05 were identified and considered for further analysis [36].

### 2.5. PPI Network Construction and Identification of Hub Genes

To systematically map the functional interactions among the DEFRDGs, a protein–protein interaction (PPI) network was constructed using the STRING database (https://string-db.org/ (accessed on 1 July 2025) [37]) and visualized with Cytoscape software (v3.10.4). Subsequently, hub genes were identified using the Cytohubba plugin. For this purpose, the top 10 genes ranked by each of six topological algorithms—MCC, MNC, Degree, DMNC, and EPC—were selected. The overlapping genes among these five sets were then defined as the final hub genes.

### 2.6. Validation of Hub Genes

We used an independent dataset, GSE52093, to validate the discovery results. The diagnostic performance of the hub genes was assessed using receiver operating characteristic (ROC) curve analysis, implemented with the ROC package (v1.18.0) in R. The curves were visualized using ggplot2 (v3.4.4) and diagnostic accuracy was measured by the area under the curve (AUC). We made the following definition: Genes with an AUC > 0.7 have diagnostic value.

### 2.7. mRNA -miRNA Regulatory Network Analysis

To understand the post-transcriptional regulatory environment of the hub genes, possible targeting miRNAs were predicted using the miRWalk online database (http://mirwalk.umm.uni-heidelberg.de/ (accessed on 1 August 2025) [38]). Only miRNA–target interactions simultaneously supported by TargetScan, miRDB, and miRTarBase with a prediction score ≥ 0.9 were retained. The regulatory network interaction between these targets and predicted miRNA regulators was constructed using the ggalluvial package (v0.12.3) in R.

### 2.8. Immune Infiltration Analysis

To characterize the immune profile of the samples, we applied two complementary algorithms to the data. First, we estimated the relative immune infiltration level of 10 immune cell types using the MCP-counter method, implemented via the MCPcounter package (v1.2.0) in R. Subsequently, the ESTIMATE algorithm, executed with the estimate package (v1.0.13), was employed to derive stromal and immune scores for each sample.

### 2.9. Histology and Immunohistochemistry

The aortic tissue specimens were fixed at room temperature in 4% paraformaldehyde for 24 h for histological examination. After paraffin-embedded sections, they were stained with hematoxylin and eosin (HE) using a high-definition isothermal staining kit (Servicebio, G1076, Wuhan, China). To evaluate collagen deposition, Masson tricolor staining was performed using the Masson staining kit (Servicebio, G1006, Wuhan, China). Elastic fiber structure was assessed using Elastic Van Gieson (EVG) staining with a commercial kit (Servicebio, G1042, Wuhan, China). For immunohistochemical analysis, paraffin-embedded sections were deparaffinized in xylene, rehydrated through a graded ethanol series, and heat-induced epitope retrieval was performed in EDTA buffer (pH 8.0). Endogenous peroxidase activity was quenched by incubation with 3% H_2_O_2_, and non-specific binding sites were blocked with 3% bovine serum albumin (BSA) at room temperature. Sections were then incubated overnight at 4 °C with primary antibodies, and subsequently incubated with horseradish peroxidase (HRP)-conjugated secondary antibodies for 50 min at room temperature. Sections were rinsed with phosphate-buffered saline (PBS) between each step. Immunoreactivity was visualized using a 3,3′-diaminobenzidine (DAB) kit. The reaction was monitored microscopically and ended by rinsing with tap water when brownish-yellow positive signals appeared. The following primary antibodies and dilutions were used: α-SMA (1:3000, 14395-1-AP, Proteintech, Wuhan, China), HIF1A (1:500, GB151339, Servicebio, Wuhan, China), TIMP1 (1:1000, GB115312, Servicebio, Wuhan, China), IL6 (1:500, GB11117, Servicebio, Wuhan, China), HMOX1 (1:500, GB15549, Servicebio, Wuhan, China), SAT1 (1:200, 10708-1-AP, Proteintech, Wuhan, China), and LPCAT3 (1:200, YT8099, Immunoway, Suzhou, China).

### 2.10. Statistical Analysis

To ensure robustness of the statistical analysis, we first tested the data for normality. For comparisons between two groups, we tested normally distributed variables using an unpaired Student’s t-test, and non-normally distributed variables using the Wilcoxon rank-sum test. A *p*-value < 0.05 was considered statistically significant.

## 3. Results

### 3.1. Overall Study Design

**Figure 1:**
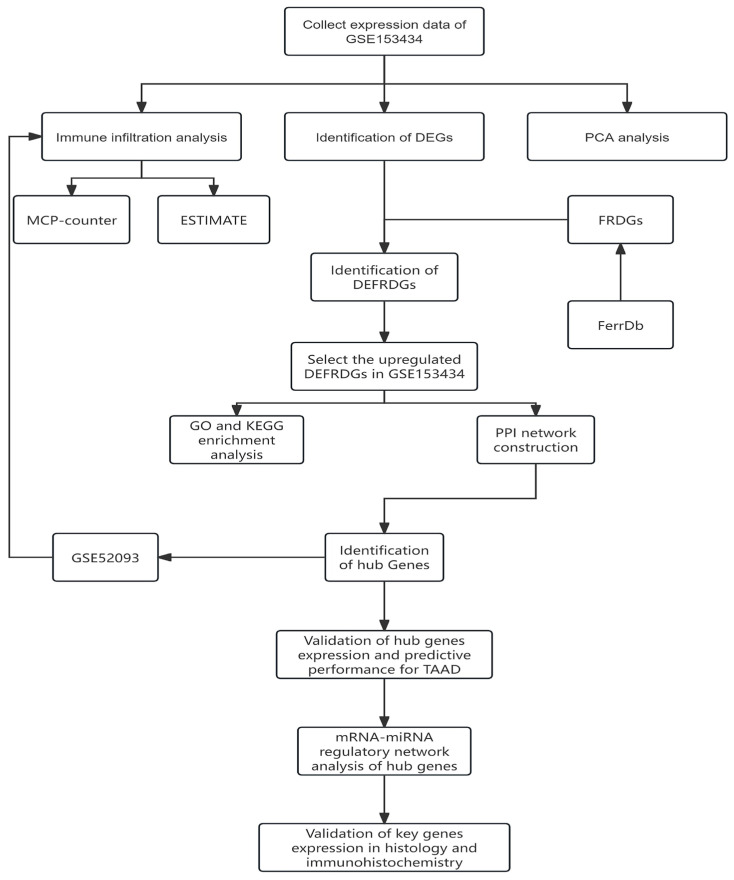
Workflow chart of the study design.

The overall flowchart of the study is illustrated in Figure 1.

### 3.2. Differential Expression Analysis of FRDGs in TAAD

**Figure 2:**
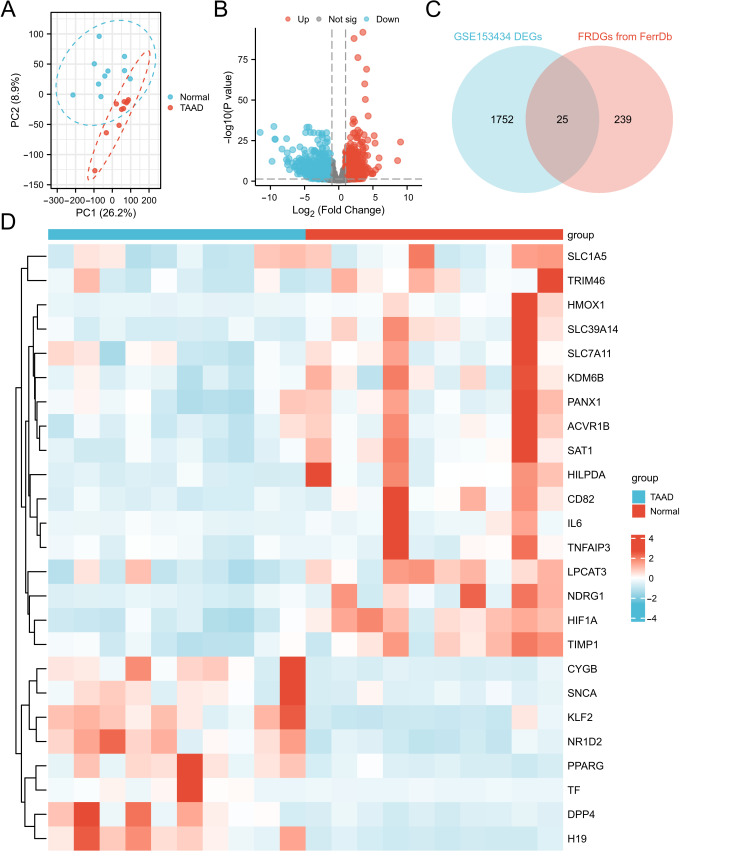
Differential expression analysis of ferroptosis-related driver genes in TAAD. (**A**) PCA of the GSE153434 dataset. (**B**) Volcano plot showing DEGs in GSE153434. (**C**) Venn diagram identifying DEFRDGs between TAAD and control samples. (**D**) Heatmap depicting the expression profiles of 25 DEFRDGs in TAAD.

To assess the reproducibility of the data within the group, PCA was performed on the GSE153434 dataset. The variance explained by PC1 was 26.2%, while PC2 accounted for 8.9%. These results confirm the good reproducibility of the GSE153434 dataset (Figure 2A). Subsequently, the GSE153434 dataset was screened using the GEO2R (https://www.ncbi.nlm.nih.gov/geo/geo2r/ (accessed on 1 May 2025) tool with differential expression criteria set at a Benjamini–Hochberg adjusted *p*-value < 0.05 and |log2Fold Change (FC)| > 1. This analysis yielded 1777 DEGs, comprising 688 upregulated and 1089 downregulated genes, visualized in a volcano plot using the R ggplot2 package (Figure 2B). A list of 264 FRDGs was obtained from the FerrDb database. Intersection of these with the TAAD-related DEGs identified 25 DEFRDGs, including 17 upregulated and 8 downregulated genes, as shown in a Venn diagram (generated with ggplot2 and VennDiagram packages; Figure 2C). A heatmap depicting the expression patterns of these 25 DEFRDGs in TAAD was generated using ggplot2 (Figure 2D).

### 3.3. GO and KEGG Enrichment Analyses and Spearman Correlation Analysis of DEFRDGs

**Figure 3:**
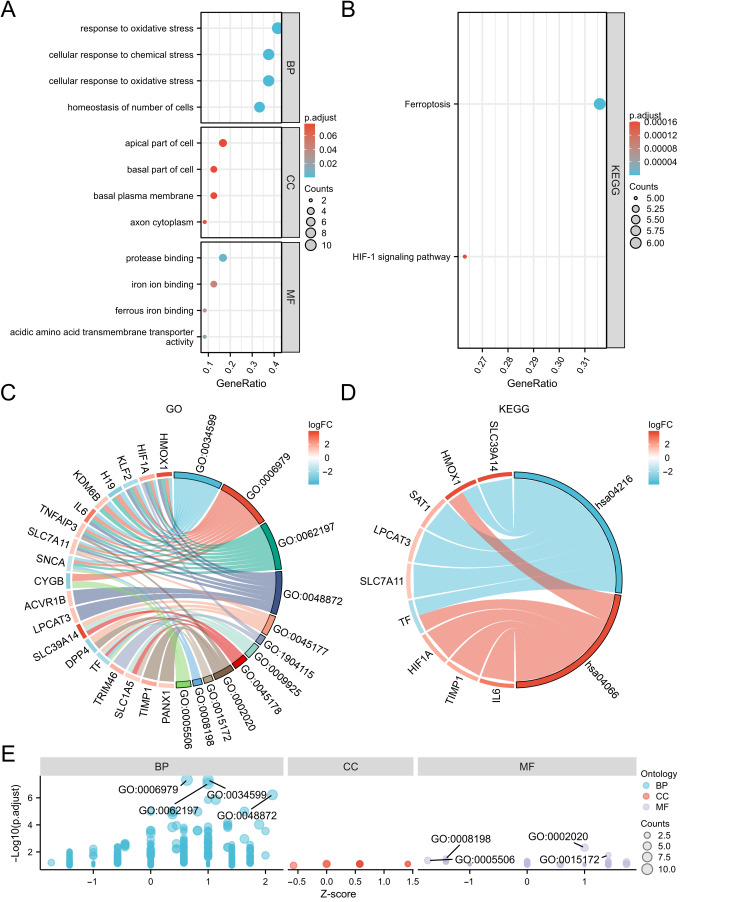
GO and KEGG enrichment analyses of DEFRDGs. (**A**) Bubble plot of GO enrichment analysis for DEFRDGs. (**B**) Bubble plot of KEGG pathway enrichment analysis for DEFRDGs. (**C**) Chord diagram illustrating GO terms enriched by DEFRDGs, integrated with logFC. (**D**) Chord diagram illustrating KEGG pathways enriched by DEFRDGs, integrated with logFC. (**E**) Bubble plot of GO enrichment analysis for DEFRDGs, integrated with logFC.

**Figure 4:**
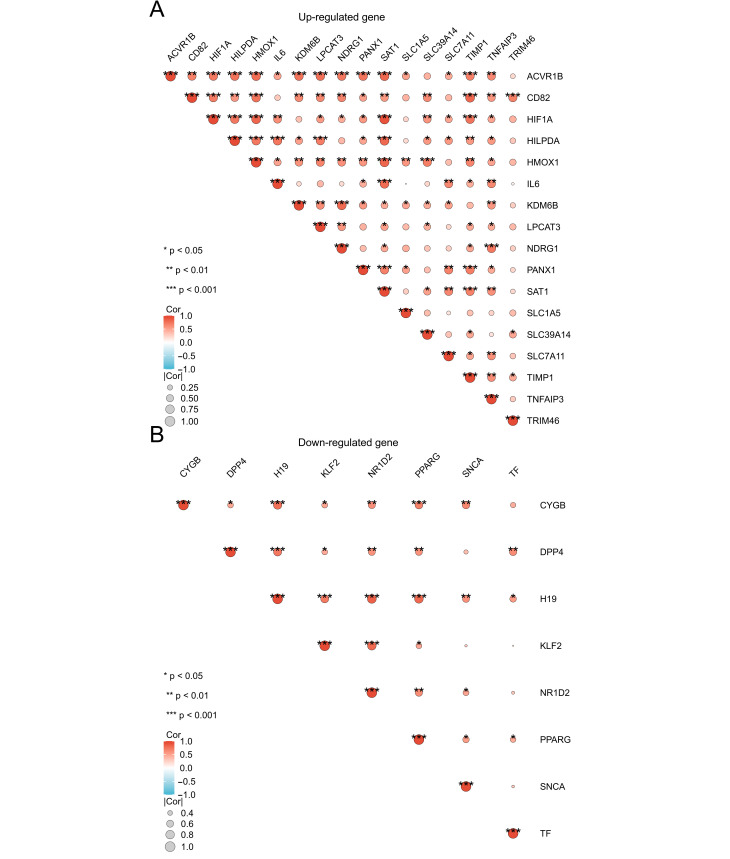
Spearman correlation analysis of DEFRDGs. (**A**) Correlation matrix of 17 upregulated DEFRDGs in TAAD. (**B**) Correlation matrix of 8 downregulated DEFRDGs in TAAD.

To explain potential biological functions of the 25 DEFRDGs, GO and KEGG enrichment analysis was performed using R software (v4.2.1). GO analysis revealed that the Biological Process (BP) terms are most enriched in ‘cellular response to oxidative stress’, ‘chemical stress’, and ‘homeostasis of cell number’. In Molecular Function (MF) terms, the DEFRDGs exhibit a high enrichment in ‘protease binding’, ‘iron ion binding’, ‘ferrous iron binding’, and ‘acidic amino acid transmembrane transporter activity’ (Figure 3A,C,E; Supplementary Tables S1 and S2). KEGG pathway analysis demonstrated significant enrichment in ‘Ferroptosis’ and the ‘HIF-1 signaling pathway’ (Figure 3B,D; Supplementary Tables S1 and S2). Furthermore, Gene Set Enrichment Analysis (GSEA) of the upregulated genes in the GSE153434 dataset shows significant enrichment of ferroptosis-related pathways, whereas enrichment among downregulated genes did not reach statistical significance (Supplementary Figure S1). Finally, Spearman correlation analysis revealed statistically significant positive and negative correlations among the 17 upregulated and 8 downregulated DEFRDGs, suggesting complex intragenic regulatory relationships (Figure 4A,B).

### 3.4. PPI Network Construction and Identification of Hub Genes

**Figure 5:**
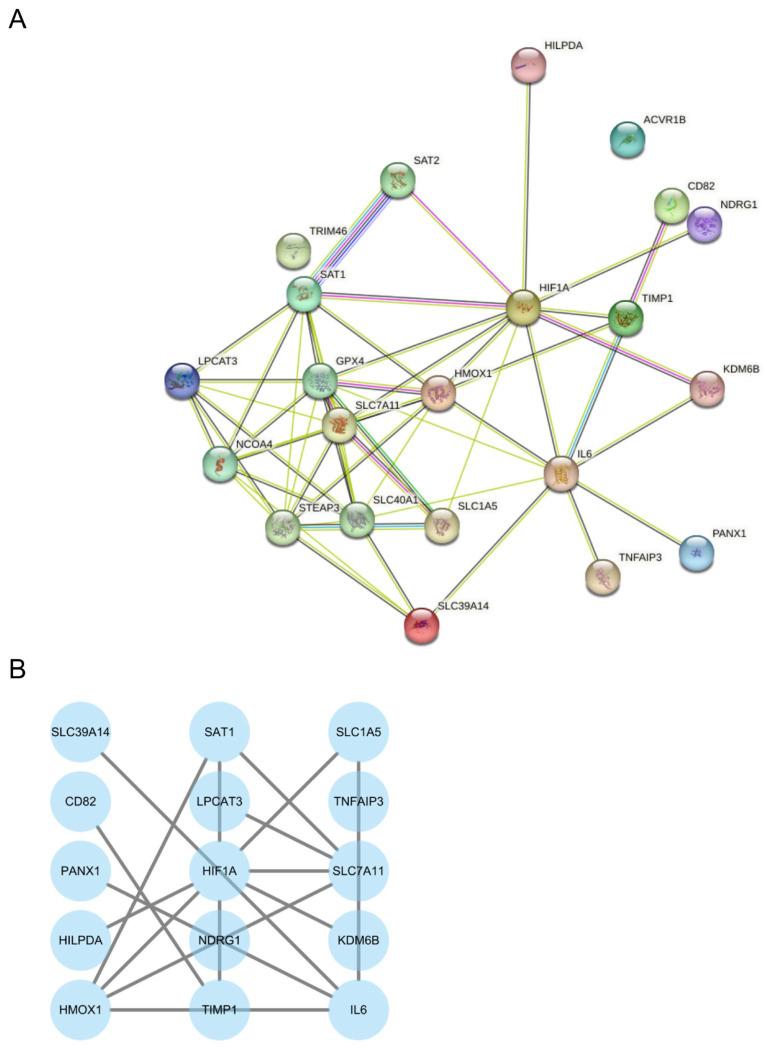
PPI network analysis. (**A**) PPI network of all DEFRDGs constructed using the STRING database. (**B**) PPI network of 25 DEFRDGs visualized with Cytoscape software (v3.10.4).

**Figure 6:**
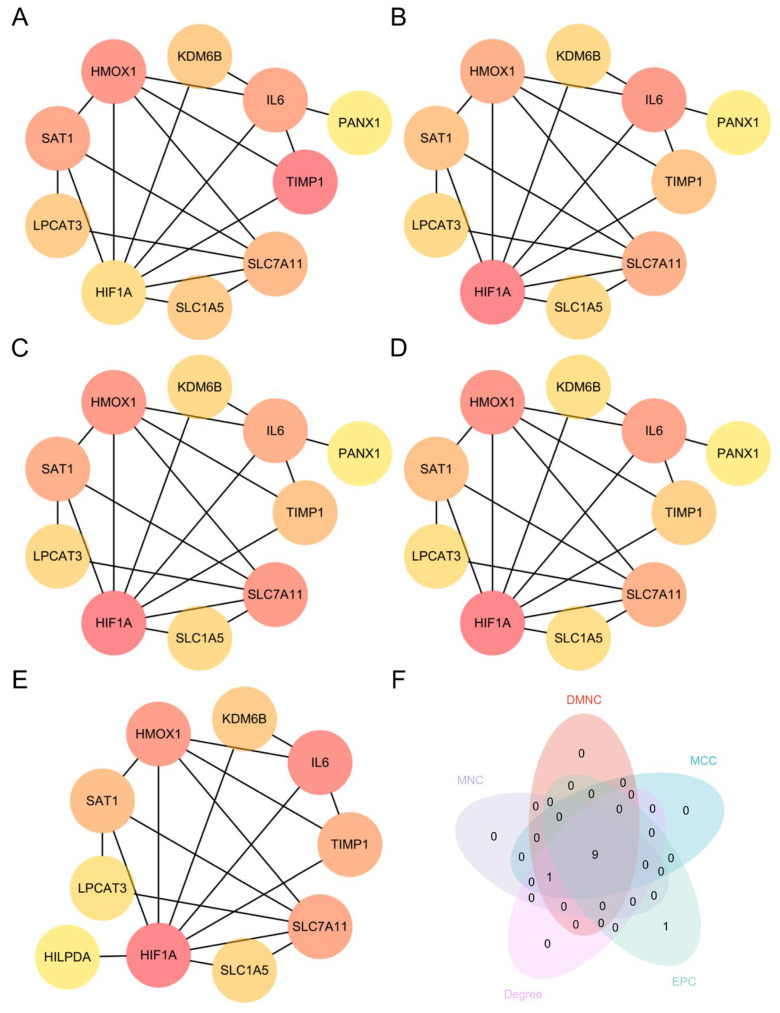
Identification of hub genes. (**A**–**E**) Top 10 genes ranked by DMNC (**A**), Degree (**B**), MCC (**C**), MNC (**D**) and EPC (**E**) algorithms. (**F**) Venn diagram showing the intersection of hub genes identified by the five algorithms.

To systematically investigate potential interactions among the 25 DEFRDGs, the PPI network was constructed using the STRING database (Figure 5A). Given that 17 of the DEFRDGs were upregulated in TAAD, subsequent PPI network construction and hub gene identification focused specifically on this subset. The network was imported into Cytoscape software (v3.10.4) for visualization and topological analysis (Figure 5B). The top 10 genes were ranked using the Cytohubba plugin by five topological algorithms: DMNC, Degree, MCC, MNC, and EPC (Figure 6A–E). The comprehensive scores for all DEFRDGs across these algorithms are provided in Supplementary Table S3. The intersection of the top 10 genes from all five algorithms yielded nine hub genes: HMOX1, HIF1A, TIMP1, IL6, LPCAT3, KDM6B, SAT1, SLC1A5, and SLC7A11 (Figure 6F).

### 3.5. Validation of Hub Genes Using the GSE52093 Dataset

**Figure 7:**
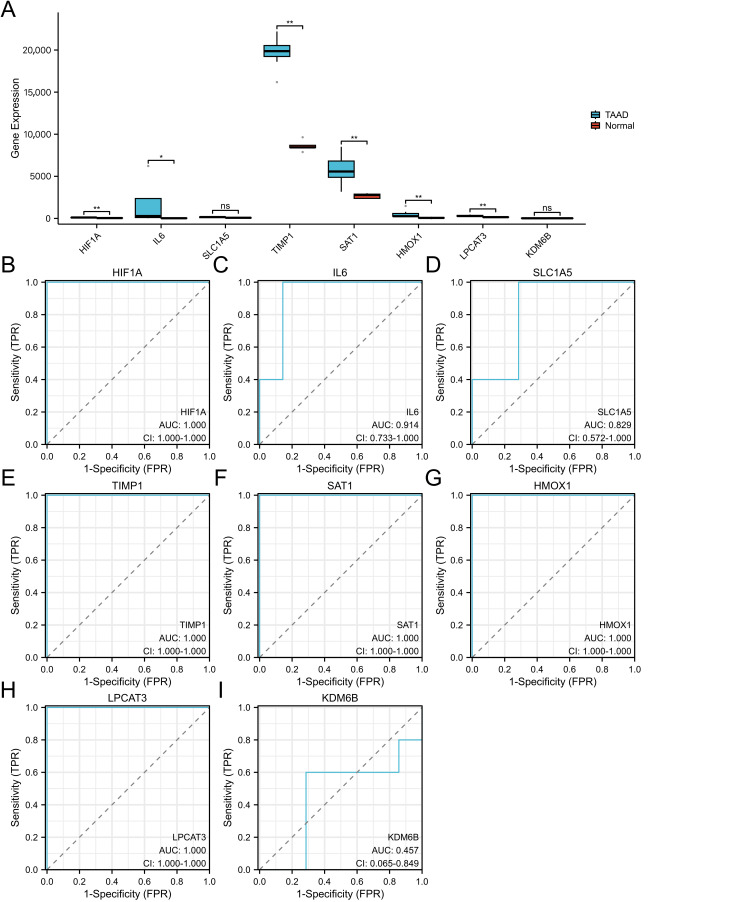
Validation of hub gene expression in the GSE52093 dataset. (**A**) Expression levels of hub genes in TAAD and normal aortic samples from GSE52093. (**B**–**I**) ROC curves evaluating the diagnostic performance of hub genes in TAAD from GSE52093. * *p* < 0.05; ** *p* < 0.01.

We used an independent GSE52093 dataset for validation. After preprocessing, expression data for eight selected hub genes were extracted and analyzed (SLC7A11 was not detected in this dataset, likely due to platform-specific probe annotation or preprocessing filtering). Among these eight, six genes (HIF1A, IL6, TIMP1, SAT1, HMOX1, and LPCAT3) were significantly upregulated in TAAD samples, consistent with the GSE153434 analysis (Figure 7A). ROC curve analysis indicated excellent predictive performance for TAAD by these six genes in GSE52093 (Figure 7B,C,E,F,G,H), suggesting their potential as diagnostic biomarkers. Notably, five genes (HIF1A, TIMP1, SAT1, HMOX1, and LPCAT3) achieved an AUC of 1.000, while IL6 also exhibited high diagnostic accuracy (AUC = 0.914). Consistent with the predefined criterion that genes with AUC > 0.7 are considered to have diagnostic value, all six genes were identified as candidate biomarkers and were subsequently included in immunohistochemical validation. In contrast, SLC1A5 and KDM6B showed no significant predictive value (Figure 7D,I).

### 3.6. Immune Infiltration Analysis

**Figure 8:**
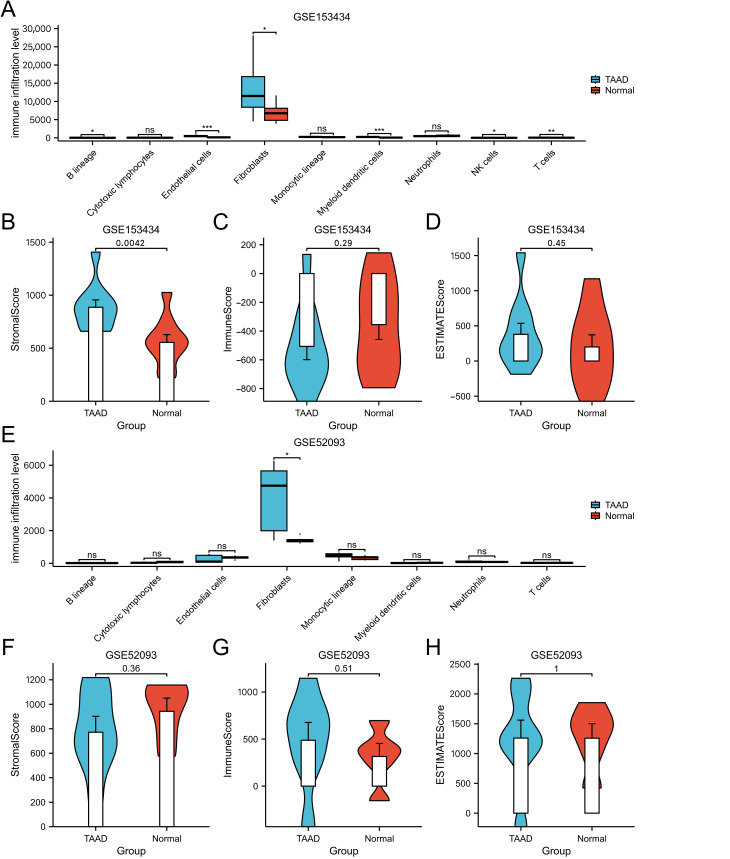
Immune infiltration analysis. (**A**) Comparison of immune cell proportions between TAAD and normal samples in the GSE153434. (**B**–**D**) Immune score and stromal score comparisons between TAAD and normal samples in GSE153434. (**E**) Comparison of immune cell proportions between TAAD and normal samples in GSE52093. (**F**–**H**) Immune score and stromal score comparisons between TAAD and normal samples in GSE52093. ns, no significant difference; *, *p* < 0.05; **, *p* < 0.01; ***, *p* < 0.001.

The relative abundance of ten immune cell types in each sample was quantified using the MCP-counter algorithm based on the gene expression profiles of the GSE153434 and GSE52093 datasets (Supplementary Table S4 for GSE153434, Supplementary Table S5 for GSE52093). Differential infiltration between TAAD and normal samples was subsequently assessed. Fibroblasts emerged as the sole immune cell type exhibiting significantly different infiltration levels consistently across both datasets (GSE153434: Figure 8A; GSE52093: Figure 8E). Further analysis of stromal and immune scores revealed dataset-specific patterns. In the GSE153434 discovery cohort, stromal scores were significantly elevated in TAAD samples, whereas immune scores showed no significant difference (Figure 8B–D). In contrast, analysis of the GSE52093 validation dataset detected no significant differences in either stromal or immune infiltration scores between TAAD and normal samples (Figure 8F–H). This discrepancy may be attributable to differences in cohort size or patient heterogeneity.

### 3.7. mRNA-miRNA Regulatory Network Analysis

The miRWalk online database was employed to predict miRNAs targeting the hub genes, leading to the construction of an mRNA-miRNA regulatory network. Potential interactions were identified, including HIF1A with miR-138-5p, miR-18b-5p, and miR-199a-5p; LPCAT3 with miR-185-5p, miR-506-3p, and miR-4644 (Supplementary Figure S2, Supplementary Table S6).

### 3.8. Immunohistochemical Validation of Key Gene Expression in Clinical Samples

**Figure 9:**
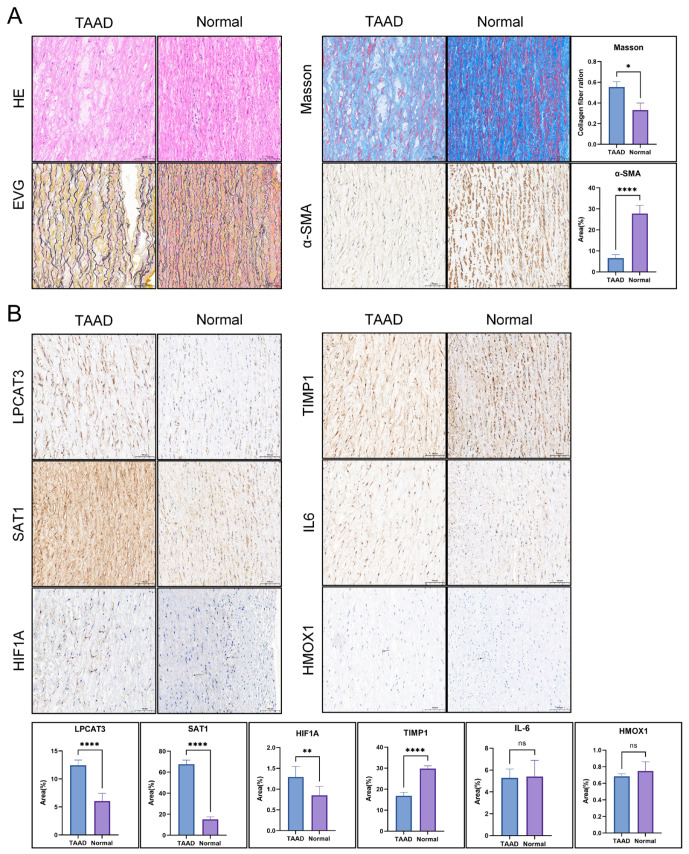
Immunohistochemical validation of key gene expression in clinical specimens. (**A**) Representative HE, EVG, and Masson’s trichrome staining showing histological alterations in TAAD and control aortic tissues. TAAD tissues exhibit fragmentation and rupture of elastic fibers in the aortic media and increased collagen deposition. α-SMA staining shows reduced expression in TAAD tissues. (**B**) Representative immunohistochemical images of HIF1A, TIMP1, SAT1, HMOX1, IL6 and LPCAT3 in TAAD and control samples. Black arrows indicate representative positive staining regions for HIF1A and HMOX1, which exhibit relatively weak and focal immunoreactivity. All images were captured at ×20 magnification. Scale bar = 100 μm. ns, no significant difference; *, *p* < 0.05; **, *p* < 0.01; ****, *p* < 0.0001.

To validate the expression patterns of candidate genes identified from our previous analyses, we collected human thoracic aortic tissues from normal controls and patients with TAAD. HE and Masson’s trichrome staining revealed distinct structural alterations in TAAD specimens. While control aortas exhibited uniformly organized elastic lamellae, TAAD tissues were characterized by disrupted elastic fiber architecture and markedly increased collagen deposition, indicative of adverse extracellular matrix remodeling. In addition, EVG staining demonstrated fragmentation and rupture of elastic fibers within the aortic media in TAAD tissues, reflecting severe structural impairment of the vessel wall. Consistently, immunohistochemical analysis showed decreased α-SMA expression in TAAD tissues, suggesting loss or phenotypic alteration of vascular smooth muscle cells (Figure 9A). Immunohistochemistry was performed to assess protein expressions of HIF1A, TIMP1, SAT1, HMOX1, IL6 and LPCAT3 (Figure 9B). As we have seen before, TAAD samples exhibit significantly higher immunoreactivity for HIF1A, SAT1 and LPCAT3 than normal controls. In contrast, TIMP1 expression was predominantly observed in normal aortic tissues, whereas HMOX1 and IL6 did not differ significantly between the two groups. Collectively, these findings implicate HIF1A, SAT1 and LPCAT3 as key contributors to TAAD pathogenesis, reinforcing the potential role of ferroptosis-related pathways in this aortic disease.

## 4. Discussion

Mounting evidence implicates ferroptosis activation in the pathogenesis of aortic dissection. A recent study identified upregulation of TNKS1 as a key regulator that activates ferroptosis and exacerbates aortic dissection formation [39]. Moreover, accumulating studies have demonstrated that ferroptosis in AD is characterized by dysregulation of iron metabolism, oxidative stress, and lipid peroxidation. For instance, iron metabolism–related proteins, including transferrin receptor (TFR), HMOX1, and ferritin, are upregulated, whereas key ferroptosis suppressors such as SLC7A11, GPX4, and FSP1 are downregulated, indicating a shift toward a pro-ferroptotic state [40]. In addition, hypoxia- and inflammation-related pathways further amplify ferroptosis. Activation of the HIF-1α/HMOX1 axis and IL-6/JAK1/STAT3 signaling has been shown to promote iron accumulation and VSMC injury, thereby accelerating AD progression [41,42]. Oxidative stress and impaired antioxidant defense, including NRF2-related pathways, also contribute to ferroptosis-mediated vascular damage [43]. Furthermore, lipid peroxidation regulators such as ACSL4 have been identified as key drivers of ferroptosis, and pharmacological interventions targeting ferroptosis pathways can alleviate AD progression [21]. Collectively, these findings suggest that ferroptosis in AD is a multifactorial process involving coordinated dysregulation of iron metabolism, hypoxia signaling, oxidative stress, and lipid peroxidation. Therefore, investigating the relationship between ferroptosis and TAAD, and specifically identifying key genes involved, is crucial for improving TAAD diagnosis and prognosis prediction.

Utilizing bioinformatics analysis coupled with clinical sample validation, this study identified key driver genes involved in ferroptosis, systematically elucidating the potential critical role of FRDGs in TAAD pathogenesis. By intersecting DEGs from the GSE153434 dataset with known ferroptosis drivers from FerrDb, we identified a molecular signature of 25 DEFRDGs in TAAD, consisting of 17 upregulated genes (SLC39A14, HMOX1, HIF1A, CD82, TIMP1, SAT1, ACVR1B, PANX1, LPCAT3, NDRG1, HILPDA, KDM6B, IL6, TNFAIP3, SLC1A5, SLC7A11, TRIM46) and 8 downregulated genes (NR1D2, KLF2, H19, DPP4, CYGB, PPARG, TF, SNCA). Subsequent bioinformatics exploration revealed that these 25 DEFRDGs are primarily involved in responses to extracellular stimuli and oxidative stress, protease and iron ion binding, and are functionally linked to the ferroptosis signaling pathway. GSEA further confirmed significant enrichment of ferroptosis-related gene sets among the upregulated genes in GSE153434. Consequently, we focused on the 17 upregulated genes for PPI network construction and hub gene identification, which pinpointed six core hub genes: HIF1A, TIMP1, SAT1, HMOX1, IL6 and LPCAT3. These genes were consistently and significantly upregulated in an independent validation cohort (GSE52093) and demonstrated exceptional diagnostic efficacy (AUC > 0.7). Immune infiltration analysis indicated a significant increase in fibroblast infiltration within TAAD tissues. Prior research has shown that fibroblast subpopulations with high LOX expression exhibit dynamic changes during aortic dissection progression [44], suggesting their potential contribution to TAAD formation, although the precise relationship remains unclear. Furthermore, miRWalk predictions identified miRNAs such as miR-138-5p, miR-18b-5p, miR-199a-5p, miR-185-5p, miR-506-3p and miR-4644 as potential regulatory elements in TAAD pathogenesis. Most importantly, immunohistochemical staining confirmed the upregulated protein expression of HIF1A, SAT1, and LPCAT3 in TAAD tissues.

LPCAT3, a member of the lysophospholipid acyltransferase (LPLAT) and membrane-bound O-acyltransferase (MBOAT) families, regulates ferroptosis by modulating the abundance of polyunsaturated phospholipids in cell membranes. It has been identified as a promising therapeutic target for cardiometabolic disorders [45] and acetaminophen-induced acute liver injury [46]. Studies have demonstrated that LPCAT3 knockdown inhibits ferroptosis [47], while its overexpression enhances cellular sensitivity to ferroptotic cell death [48], positioning LPCAT3 as a potential diagnostic and therapeutic target for TAAD.

SAT1, a key enzyme in polyamine metabolism, acetylates polyamines to regulate intracellular polyamine homeostasis and redox balance [49]. Evidence suggests SAT1 promotes osteoarthritis progression by activating ferroptosis signaling [49], and it plays roles in triple-negative breast cancer [50] and ovarian cancer [51]. Its specific function in TAAD warrants further investigation.

HIF1A plays a crucial role as a transcriptional subunit in facilitating cellular responses to low oxygen levels within tissues. Its involvement has been linked to the development of several cardiovascular conditions [52]. This is the result of high expression of HIF1A in TAAD [53]. Notably, recent studies suggest that HIF1A can promote ferroptosis through regulation of iron metabolism and oxidative stress pathways, including activation of the HMOX1 axis [54]. Collectively, these multi-level lines provide a molecular framework for FRDGs in TAAD and provide new insights into complex mechanisms of this disease.

The combined body of research forms a detailed molecular model focused on FRDGs in the context of TAAD pathology. This framework offers fresh perspectives on the intricate pathways contributing to the onset and progression of this particular disease.

## 5. Conclusions

In this study, a panel of DEFRDGs was identified between normal aortic samples and those from patients with TAAD. Enrichment analysis indicated involvement in cellular responses to external stimuli and oxidative stress, protease, iron, and ferrous ion binding. Notably, they were significantly associated with the ferroptosis and HIF-1 signaling pathways, implicating a potential mechanistic role for ferroptosis in TAAD pathogenesis. Through integrated bioinformatic analysis, nine hub genes were subsequently identified as critical DEFRDGs in TAAD. ROC curve analysis, together with immunohistochemical and immunofluorescence validation in clinical tissue samples, confirmed that three of these genes—LPCAT3, SAT1, and HIF1A—exhibited statistically significant differential expression. These findings may offer novel insights into the molecular underpinnings of TAAD and suggest potential diagnostic biomarkers or therapeutic targets. Furthermore, prediction of miRNA interactions revealed several candidate miRNAs potentially regulating these hub genes, including miR-138-5p, miR-18b-5p, miR-199a-5p, miR-185-5p, miR-506-3p and miR-4644. Immune Infiltration revealed that changes in fibroblast abundance may contribute to TAAD pathogenesis. Conclusion, we suggest that LPCAT3, SAT1 and HIF1A may play crucial roles in TAAD through the ferroptosis and hypoxia pathway. However, further experimental studies are warranted to validate the expression patterns and the precise functional roles of these genes.

## Data Availability

Data can be downloaded from the online public databases GEO.

## Supplementary Materials

The following supporting information can be downloaded at: https://www.mdpi.com/article/10.3390/cimb48040382/s1.

## Abbreviations

The following abbreviations are used in this manuscript:

AD	Aortic dissection
AUC	Area under the curve
BP	Biological Process
DAB	Diaminobenzidine
DEFRDGs	Differentially expressed ferroptosis-related driver genes
DEGs	Differentially expressed genes
ECM	Extracellular matrix
EVG	Elastic Van Gieson
FRDGs	Ferroptosis-related driver genes
GEO	Gene Expression Omnibus
GO	Gene Ontology
GSEA	Gene Set Enrichment Analysis
HE	Hematoxylin and eosin
HRP	Horseradish peroxidase
KEGG	Kyoto Encyclopedia of Genes and Genomes
MF	Molecular Function
PBS	Phosphate-buffered saline
PCA	Principal Component Analysis
PPI	Protein–protein interaction
ROC	Receiver operating characteristic
TAAD	Stanford type A aortic dissection
TFR	Transferrin receptor
VSMCs	Vascular smooth muscle cells
