# Supplementary material for: Bioinformatics Analysis of Ferroptosis-Related Driver Genes in Stanford Type A Aortic Dissection
Source: Curr Issues Mol Biol. 2026 Apr 7;48(4):382. doi: 10.3390/cimb48040382 (PMC13114551; doi:10.3390/cimb48040382)
Supplement: Supplementary file 1 [file cimb-48-00382-s001.zip › Supplementary Table S1.pdf]

Supplementary Table 1. GO and KEGG enrichment analysis of DEFRDGs

Ontology	ID	Description	GeneRatio	BgRatio	pvalue	p.adjust
BP	GO:0034599	cellular response to oxidative stress	9/24	284/18800	3.87e-11	4.81e-08
BP	GO:0006979	response to oxidative stress	10/24	433/18800	5.57e-11	4.81e-08
BP	GO:0062197	cellular response to chemical stress	9/24	332/18800	1.55e-10	8.95e-08
BP	GO:0048872	homeostasis of number of cells	8/24	282/18800	1.39e-09	5.99e-07
BP	GO:0000302	response to reactive oxygen species	7/24	203/18800	4.57e-09	1.39e-06
CC	GO:0045177	apical part of cell	4/24	424/19594	0.0016	0.0773
CC	GO:1904115	axon cytoplasm	2/24	62/19594	0.0026	0.0773
CC	GO:0009925	basal plasma membrane	3/24	251/19594	0.0034	0.0773
CC	GO:0045178	basal part of cell	3/24	269/19594	0.0042	0.0773
CC	GO:0120111	neuron projection cytoplasm	2/24	89/19594	0.0053	0.0773
MF	GO:0002020	protease binding	4/24	136/18410	2.7e-05	0.0048
MF	GO:0015172	acidic amino acid transmembrane transporter activity	2/24	16/18410	0.0002	0.0173
MF	GO:0008198	ferrous iron binding	2/24	26/18410	0.0005	0.0310
MF	GO:0005506	iron ion binding	3/24	151/18410	0.0010	0.0432
MF	GO:0015175	neutral amino acid transmembrane transporter activity	2/24	41/18410	0.0013	0.0464
KEGG	hsa04216	Ferroptosis	6/19	41/8164	2.83e-10	2.35e-08
KEGG	hsa04066	HIF-1 signaling pathway	5/19	109/8164	3.88e-06	0.0002
