# Supplementary material for: Bioinformatics Analysis of Ferroptosis-Related Driver Genes in Stanford Type A Aortic Dissection
Source: Curr Issues Mol Biol. 2026 Apr 7;48(4):382. doi: 10.3390/cimb48040382 (PMC13114551; doi:10.3390/cimb48040382)
Supplement: Supplementary file 1 [file cimb-48-00382-s001.zip › Supplementary Table S3.pdf]

Supplementary Table 3. DEFRDGs screened by five methods of cytoHubba in the Cytoscape software package

DMNC	Degree	MCC	MNC	EPC
HMOX1	HMOX1	HMOX1	HMOX1	NDRG1
HIF1A	HIF1A	HIF1A	HIF1A	HMOX1
TIMP1	TIMP1	TIMP1	TIMP1	HIF1A
IL6	IL6	IL6	IL6	TIMP1
LPCAT3	LPCAT3	LPCAT3	LPCAT3	IL6
PANX1	PANX1	PANX1	PANX1	LPCAT3
KDM6B	KDM6B	KDM6B	KDM6B	KDM6B
SAT1	SAT1	SAT1	SAT1	SAT1
SLC1A5	SLC1A5	SLC1A5	SLC1A5	SLC1A5
