# Supplementary figures and images for: Bioinformatics Analysis of Ferroptosis-Related Driver Genes in Stanford Type A Aortic Dissection
Source: Curr Issues Mol Biol. 2026 Apr 7;48(4):382. doi: 10.3390/cimb48040382 (PMC13114551; doi:10.3390/cimb48040382)

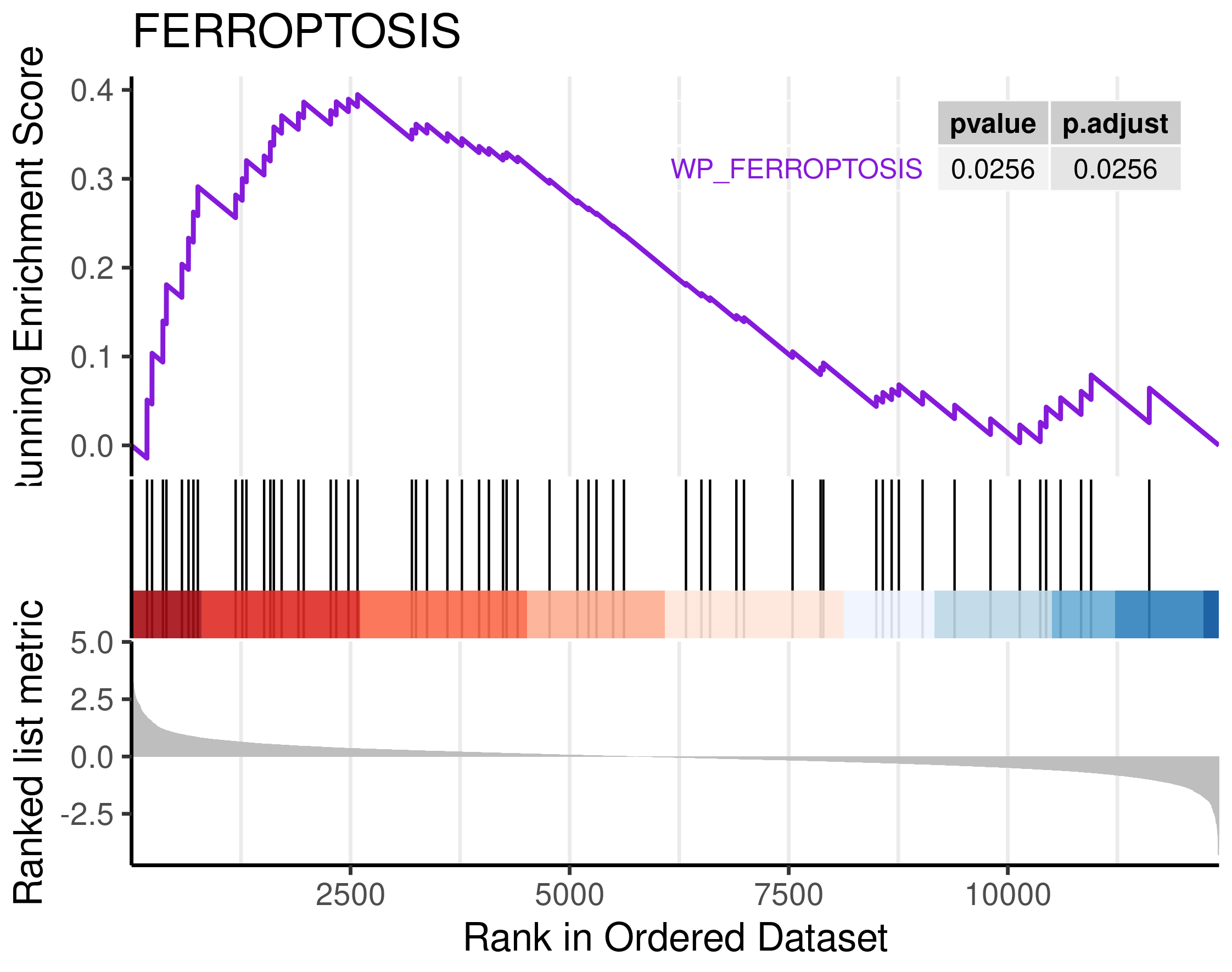

Supplement: Supplementary file 1 [file cimb-48-00382-s001.zip › Supplementary Figure S1.tiff]

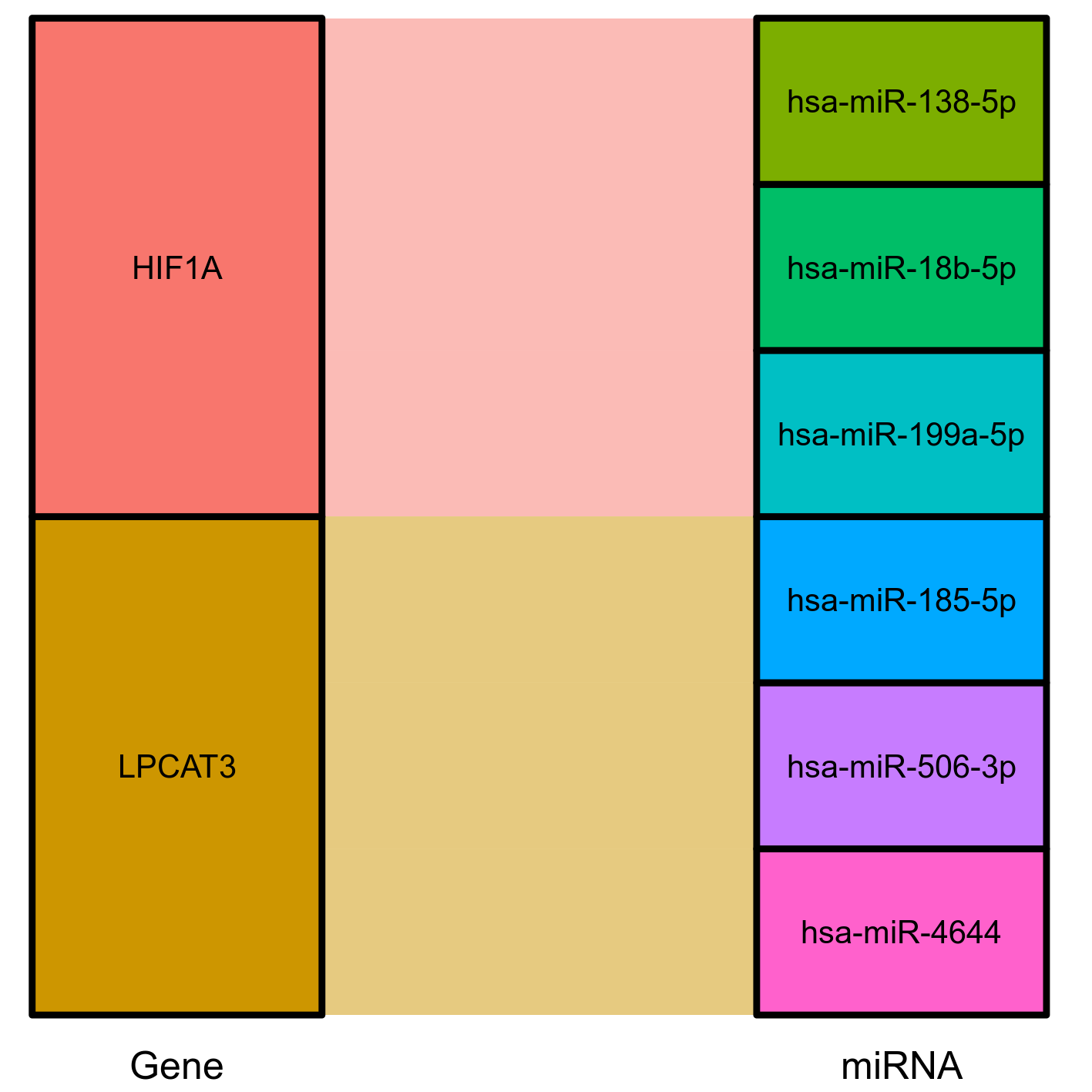

Supplement: Supplementary file 1 [file cimb-48-00382-s001.zip › Supplementary Figure S2.tiff]
